# LINC00467 induces melanoma deterioration by targeting miR-485-5p/p21 activated kinase 1

**DOI:** 10.5937/jomb0-39708

**Published:** 2023-03-15

**Authors:** Zhoujing Ji, Jie Zhang, Lili Zhang, Shengju Yang, Yangcheng Li, Lixiong Gu

**Affiliations:** 1 Affiliated Hospital of Nantong University, Department of Dermatology, Nantong, China; 2 Tumor Hospital Affiliated to Nantong University, Nantong Tumor Hospital, Department of General Surgery, Nantong, China

**Keywords:** melanoma, LINC00467, MiR-485-5p, PAK1, melanom, LINC00467, MiR-485-5p, PAK1

## Abstract

**Background:**

The purpose of the current research was to investigate the biological roles of LINC00467 in inducing melanoma deterioration.

**Methods:**

Differential level of LINC00467 in melanoma tissues and its prognostic value were analyzed in GEPIA, which were further confirmed in clinical samples we collected. Regulatory effects of LINC00467 on proliferation, migration and invasion capacities of A375 and SKMEL1 cell lines were examined by a series of functional experiments. Potential downstream targets of LINC00467 were identified through dual-luciferase reporter assay, and their synergistic role in melanoma process was finally explored by rescue experiments.

**Results:**

LINC00467 was up-regulated in melanoma samples, but it did not have a prognostic potential in melanoma. LINC00467 has the capacities to stimulate proliferation, migration and invasion of A375 and SKMEL1 cell lines. The feedback loop LINC00467/miR-485-5p/PAK1 was identified, which was responsible for inducing melanoma deterioration.

**Conclusions:**

LINC00467 stimulates proliferation, migration and invasion capacities of melanoma via targeting miR-485-5p to upregulate PAK1, which provides potential targets for treatment of melanoma.

## Abstract (sr)

**Uvod:**

Svrha trenutnog istraživanja je bila da se ispita biološka uloga LINC00467 u izazivanju pogoršanja stanja melanoma.

**Metode:**

Diferencijalni nivo LINC00467 u tkivima melanoma i njegova prognostička vrednost analizirani su u GEPIA, što je dalje potvrđeno u kliničkim uzorcima koje smo prikupili. Regulatorni efekti LINC00467 na kapacitete proliferacije, migracije i invazije A375 i SKMEL1 ćelijskih linija ispitani su nizom funkcionalnih eksperimenata. Potencijalni nizvodni ciljevi LINC00467 identifikovani su kroz test reportera sa dvostrukom luciferazom, a njihova sinergistička uloga u procesu melanoma je konačno istražena eksperimentima spasavanja.

**Rezultati:**

LINC00467 je bio pojačano regulisan u uzorcima melanoma, ali nije imao prognostički potencijal kod melanoma. LINC00467 ima kapacitete da stimuliše proliferaciju, migraciju i invaziju A375 i SKMEL1 ćelijskih linija. Identifikovana je povratna sprega LINC00467/miR-485-5p/PAK1, koja je odgovorna za izazivanje pogoršanja melanoma.

**Zaključak:**

LINC00467 stimuliše kapacitete proliferacije, migracije i invazije melanoma putem ciljanja miR-485-5p da bi se pojačao PAK1, koji pruža potencijalne mete za lečenje melanoma.

Uvod: Svrha trenutnog istra`ivanja je bila da se ispita biolo{ka uloga LINC00467 u izazivanju pogor{anja stanja melanoma.

Metode: Diferencijalni nivo LINC00467 u tkivima melanoma i njegova prognosti~ka vrednost analizirani su u GEPIA, {to je dalje potvr|eno u klini~kim uzorcima koje smo prikupili. Regulatorni efekti LINC00467 na kapacitete proliferacije, migracije i invazije A375 i SKMEL1 }elijskih linija ispitani su nizom funkcionalnih eksperimenata. Potencijalni nizvodni ciljevi LINC00467 identifikovani su kroz test reportera sa dvostrukom luciferazom, a njihova sinergi-sti~ka uloga u procesu melanoma je kona~no istra`ena eksperimentima spasavanja. Rezultati: LINC00467 je bio poja~ano regulisan u uzorcima melanoma, ali nije imao prognosti~ki potencijal kod melanoma. LINC00467 ima kapacitete da stimuli{e proliferaciju, migraciju i invaziju A375 i SKMEL1 }elijskih linija. Identifikovana je povratna sprega LINC00467/miR-485-5p/PAK1, koja je odgovorna za izazivanje pogor{anja melanoma.

Zaklju~ak: LINC00467 stimuli{e kapacitete proliferacije, migracije i invazije melanoma putem ciljanja miR-485-5p da bi se poja~ao PAK1, koji pru`a potencijalne mete za le~enje melanoma.

## Introduction

Melanoma is a highly aggressive malignancy that develops from uncontrollably proliferated melanocytes. It mainly occurs in young and middleaged people. Melanoma usually affects skin, but it can also involve the mouth, intestines, or ocular choroid [1] [2]. Melanoma is characterized by high degree of malignancy and rapid progression. Current treatment of melanoma is not ideal. Lymphatic or distant metastasis of melanoma develops in the early phase, leading to a poor prognosis with less than 20% of 5-year survival [3] [4]. So far, melanoma is still incurable, and its molecular mechanism remains largely unclear.

LncRNAs are over 200 nucleotides long, noncoding RNAs. Although they cannot encoded proteins, lncRNAs exert diverse biological functions in the form of RNAs [5] [6]. LncRNAs participate in cell functions through transcriptionally, post-transcriptionally or epigenetically regulating gene expressions. They are promising biomarkers for cancer diseases [7] [8] [9]. Screening melanoma-associated lncRNAs and clarifying their potential functions contribute to develop effective targeted therapy.

We previously analyzed the differential level of LINC00467 in melanoma tissues, which was abnormally up-regulated. In the current study, the potential functioned of LINC00467 in regulating melanoma cell phenotypes was explored.

## Materials and methods

### Melanoma profile

A melanoma profile was analyzed based on the database of GEPIA (http://gepiacancer-pku.cn/index.html). Differential level of LINC00467 and its correlation to the survival data in melanoma patients were also analyzed.

### Sample collection

Samples of melanoma and paired normal skin tissues were collected from 30 melanoma patients in our hospital from January 2015 to December 2021. Tumor staging was determined based on UICC criteria. None of the enrolled patients received anticancer therapy before surgery that was pathologically proven. Each patient provided informed consent before the study. The present study was approved by the Ethical Committee of Affiliated Hospital of Nantong University.

### Cell culture

NHEM and melanoma cell lines (SKMEL1, A375, A2058 and A875) were purchased from ATCC and were cultivated in Dulbecco's Modified Eagle Medium (DMEM) according to the manufacture's protocols.

### Transfection

Cells grew to 70% density and were then transfected by Lipo 3000. The transfection efficiency of the cells was observed under a microscope 48 hours after transfection. Transfected cells were used for further functional phenotype experiments.

### qRT-PCR

RNA from tissue samples is extracted and purified by TRIzol treatment, and the purified RNA is reverse transcribed into cDNAs. Relative expression of PCR products was calculated by 2^-ΔΔCt^ and normali zed to that of GAPDH. The primers used were listed as follows: LINC00467: Forward: 5'-ATTGAAGATGCTGCCAAGGG-3'; Reverse: 5'-GCCCAGTTTCAGTCCCTCTT-3'; MiR-485-5p: Forward: 5'-CCAAGCTTCACCCATTCCTAACAGGAC-3'; Reverse: 5'-CGGGATCCGTAGGTCAGTTACATGCATC-3'; PAK1: Forward: 5'-AAGACATCCAACAGCCAGAA-3'; Reverse: 5'-TGTAGCCACGTCCCGAGT-3'; GAPDH: Forward: 5'-GGAGCGAGATCCCTCCAAAAT-3'; Reverse: 5'-GGCTGTTGTCATACTTCTCATGG-3'; U6: Forward: 5'-GCTGAGGTGACGGTCTCAAA-3'; Reverse: 5'-GCCTCCCAGTTTCATGGACA-3'.

### EdU assay

Cells were treated with 5-ethynyl-2'-deoxyuridine (EdU) for 2 h after cell adhesion, followed by being stained with AdoLo and 4',6-diamidino-2-phenylindole (DAPI). After being washed by PBS for twice, the EdU-labeled cells were observed under a microscope.

### Transwell migration assay

A 24-well plate was taken, and 500-600 μL of culture medium for each group was added to the lower layer, and a Transwell chamber was placed on the top. After digestion, cells were resuspended with serum-free DMEM to determine cell density, and 200 μL of the desired density of cell suspension was added to each chamber and incubated for 24-72h. Aspirate the culture medium from the upper and lower chambers and add 500 μL paraformaldehyde (Xavier) to the lower chamber. After 48 h of penetration, cells were fixed, stained, and each sample was captured and counted in five random fields. 

### Dual-luciferase reporter assay

Binding sequences were predicted using online software for generating luciferase vectors. The Luciferase activity of the seeded cells was determined 48 h after the transfection.

### Statistical analysis

SPSS 17.0 (SPSS Inc., Chicago, IL, USA) was adopted for statistical analyses. Survival was evaluated using Kaplan-Meier. P<0.05 indicates statistically significant difference.

## Results

### LINC00467 was up-regulated in melanoma

**Figure 1:**
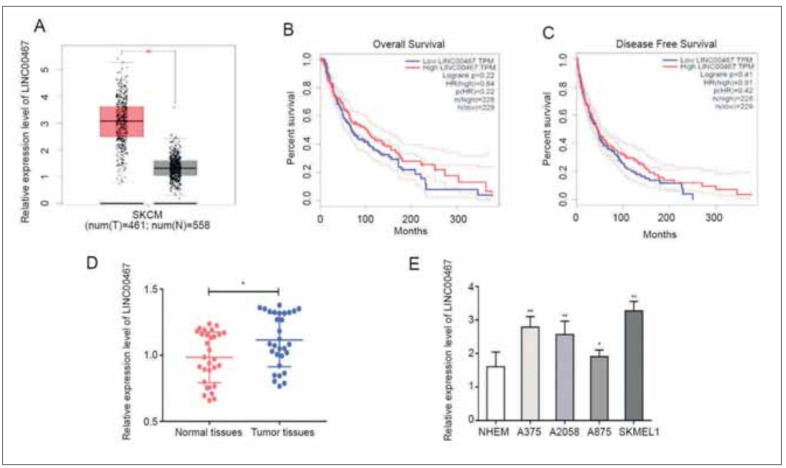
LINC00467 was up-regulated in melanoma. (A) LINC00467 levels in melanoma profile downloaded from GEPIA; (B) Correlation between LINC00467 and overall survival in melanoma; (C) Correlation between LINC00467 and disease-free survival in melanoma; (D) LINC00467 levels in melanoma tissues and normal ones; (E) LINC00467 levels in melanoma cell lines. *P<0.05; **P<0.01.

Through analyzing melanoma profile downloaded from GEPIA, LINC00467 was up-regulated in melanoma tissues than normal skin tissues (Figure 1A). However, LINC00467 did not have a correlation with the survival data of melanoma (Figure 1B-Figure 1C). Consistently, in melanoma clinical samples collected in our hospital, LINC00467 was up-regulated (Figure 1D). In vitro level of LINC00467 was highly expressed as well (Figure 1E). LINC00467 was of significance in melanoma process.

### LINC00467 stimulated proliferative, migratory and invasive functions of melanoma

**Figure 2:**
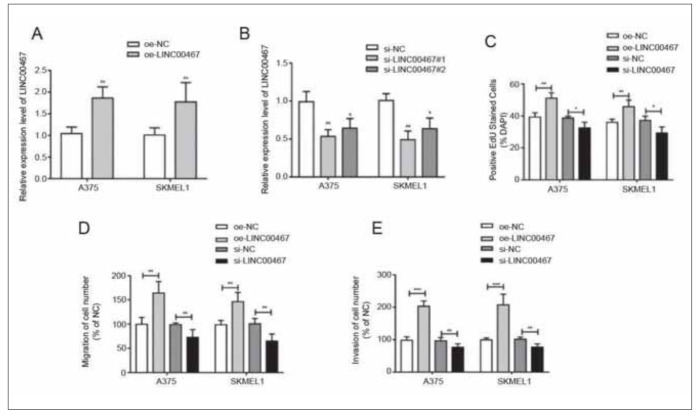
LINC00467 stimulated proliferative, migratory and invasive functions of melanoma. (A) Transfection efficacy of oe-LINC00467 in A375 and SKMEL1 cells; (B) Transfection efficacy of si-LINC00467 in A375 and SKMEL1 cells; (C) EdU-stained A375 and SKMEL1 cells regulated by LINC00467; (D) Migration in A375 and SKMEL1 cells regulated by LINC00467; (E) Invasion in A375 and SKMEL1 cells regulated by LINC00467. *P<0.05; **P<0.01; ***P<0.001.

In A375 and SKMEL1 cells, LINC00467 level was intervened by transfection of oe-LINC00467 or si-LINC00467 (Figure 2A, Figure 2B). EdU assay uncovered that overexpression of LINC00467 enhanced EdU-stained cell ratio in melanoma cells, which was reduced, conversely, by knockdown of LINC00467 (Figure 2C). Both migratory cell rate and invasive cell rate were enhanced in melanoma cells overexpressing LINC00467. Knockdown of LINC00467, on the contrary, reduced metastatic rate (Figure 2D, Figure 2E). Therefore, LINC00467 served as an oncogene in melanoma process.

### LINC00467 competitively bound miR-485-5p

**Figure 3:**
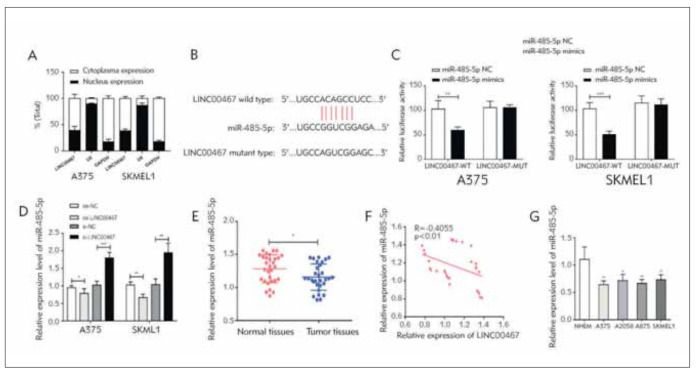
LINC00467 competitively bound miR-485-5p. (A) Subcellular distribution of LINC00467 in A375 and SKMEL1 cells; (B) Binding sequences in 3’UTR of LINC00467 and miR-485-5p; (C) Direct binding association of LINC00467 with miR-485-5p; (D) MiR-485-5p level in A375 and SKMEL1 cells regulated by LINC00467; (E) MiR-485-5p levels in melanoma tissues and normal ones; (F) A negative association of LINC00467 with miR-485-5p; (G) MiR-485-5p levels in melanoma cell lines. *P<0.05; **P<0.01; ***P<0.001.

The subcellular distribution of LINC00467 was analyzed. The results indicated that LINC00467 was mainly located at the cytoplasm of A375 and SKMEL1 cells, suggesting its post-transcriptional role (Figure 3A). Using Starbase, miR-485-5p was predicted to be a potential target of LINC00467 (Figure 3B). Later, their binding relationship was further verified (Figure 3C). Results showed that miR-485-5p expression was negatively regulated by LINC00467 (Figure 3D). In comparison to normal skin tissues, expression of miR-485-5p was significantly lower in melanoma tissues (Figure 3E). In addition, the negative association of LINC00467 with miR-485-5p in melanoma tissues was also assessed (Figure 3F). The in vitro abundance of miRNA-485-5p was identically down-regulated in melanoma cell lines (Figure 3G).

### MiR-485-5p targeted PAK1

**Figure 4:**
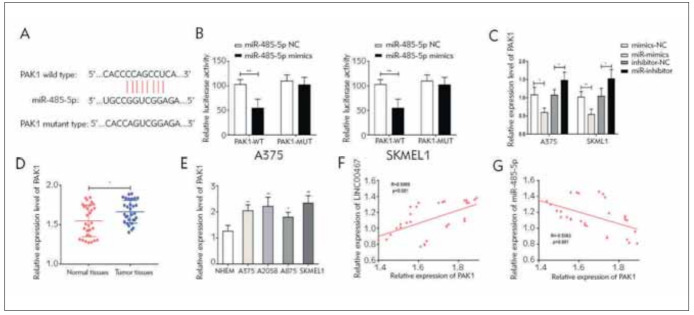
PAK1 was the target gene of miR-485-5p. (A) Binding sequences in 3’UTR of miR-485-5p and PAK1; (B) Direct binding relationship between miR-485-5p and PAK1; (C) PAK1 level in A375 and SKMEL1 cells regulated by miR-485-5p; (D) PAK1 levels in melanoma tissues and normal ones; (E) PAK1 levels in melanoma cell lines; (F) A positive correlation between LINC00467 and PAK1; (G) A negative correlation between miR-485-5p and PAK1. *P<0.05; **P<0.01.

Likewise, (P21 (RAC1) Activated Kinase 1) PAK1 was verified as the target gene of miR-485-5p (Figure 4A, Figure 4B). Our findings demonstrated that miR-485-5p negatively regulates PAK1 levels (Figure 4C). QRT-PCR data suggested that level of PAK1 remarkably increased in melanoma cells and tissues (Figure 4D, Figure 4E). It was positively correlated to LINC00467, but negatively correlated to miR-485-5p (Figure 4F, Figure 4G).

### LINC00467 regulated melanoma cell functions by mediating PAK1

**Figure 5:**
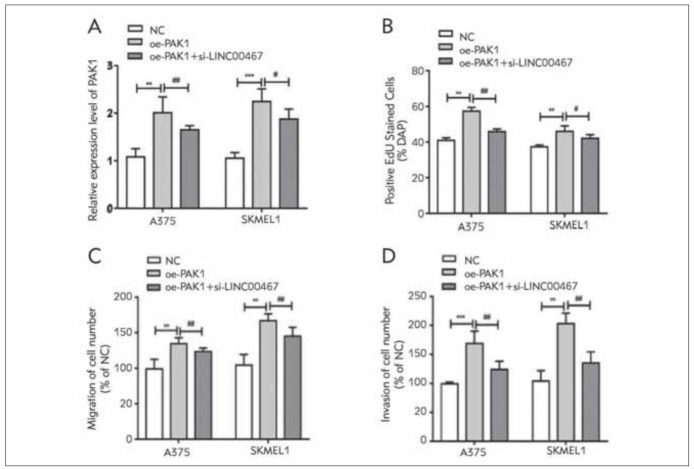
LINC00467 regulated melanoma cell functions by mediating PAK1. (A) PAK1 level in A375 and SKMEL1 cells coregulated by PAK1 and LINC00467; (B) EdU-stained A375 and SKMEL1 cells co-regulated by PAK1 and LINC00467; (C) Migration in A375 and SKMEL1 cells co-regulated by PAK1 and LINC00467; (D) Invasion in A375 and SKMEL1 cells co-regulated by PAK1 and LINC00467. **P<0.01 and ***P<0.001, vs. NC group; #P<0.05 and ##P<0.01, vs. oe-PAK1 group.

In A375 and SKMEL1 cells overexpressing PAK1, the up-regulated PAK1 could be inhibited by knockdown of LINC00467 (Figure 5A). Overexpression of PAK1 in melanoma cells remarkably enhanced EdU-stained cell ratio, migratory cell rate and invasive cell rate. However, their enhanced rates were all reversed by knockdown of LINC00467 (Figure 5B-Figure 5D). Therefore, PAK1 was responsible for LINC00467-induced melanoma deterioration.

## Discussion

Malignant melanoma is derived from melanocytes and it is the most aggressive and deadly skin cancer [10] [11]. The global prevalence of melanoma has gradually increased, which seriously endangers human lives and poses a huge economic burden [12]. Given above, it's important to explore the molecular mechanism of melanoma.

Previous research have identified the vital function of LINC00467 in several cancers [13] [14] [15] [16]. Using GEPIA database, LINC00467 was identified to be upregulated in melanoma tissues, and its up-regulated expression pattern was further confirmed in clinical samples of melanoma and cell lines. Nevertheless, LINC00467 was uncorrelated to the survival data of melanoma. *In vitro *experimentations showed that LINC000467 was capable of stimulating proliferation, migration and invasion capacities of melanoma cell lines. Since LINC00467 mainly located at the cytoplasm, we believed that it may exert a vital role in post-transcriptional regulation as a ceRNA.

The ceRNA theory proposed that circRNAs, lnc-RNAs, mRNAs, pseudogenes and other transcripts can competitively bind miRNAs through miRNA res pon se elements (MREs), thus further regulate the downstream targets of miRNAs [17] [18]. It is a novel mechanism for explaining RNA interaction. Through bio informatic prediction and functional analysis, miR-485-5p was discovered to be a possible target of LINC00467. 

PAK kinase can be activated by RAS, RacI and Cdc42 through GTPase-dependent or non-GTPase-dependent pathways. PAK is widely expressed in tissues, and its activation triggers various life activities. The cancer-associated role of PAK1 has emerged [19] [20] [21] [22]. Here, PAK1 was found to be responsible for the carcinogenic role of LINC00467 in melanoma. Taken together, a novel feedback loop was identified that LINC00467/miR-485-5p/PAK1 induced melanoma deterioration.

Several limitations should be pointed out. Firstly, only 30 clinical samples of melanoma were collected. More melanoma cases should be recruited in the future to analyze the clinical significance of LINC00467. Secondly, an *in vivo* experiment requires to be designed to validate our findings.

## Conclusions

LINC00467 stimulates proliferation, migration and invasion capacities of melanoma through targeting miR-485-5p to upregulate PAK1, which is a possible therapeutic target for melanoma.

## Dodatak

### Funding Acknowledgements

This study was supported by Nantong Science and Technology Bureau (Peripheral blood dynamic NLR and prognostic nutrition index combined with tumor thickness could guide perioperative treatment and predict prognosis of cutaneous melanoma; Project number: JCZ20211).

### Conflict of interest statement

All the authors declare that they have no conflict of interest in this work.
